# Development of a 6-Year Sarcopenia Risk Prediction Model Based on an Elderly Population: A Prospective Cohort Study

**DOI:** 10.1093/geroni/igaf122.3914

**Published:** 2025-12-31

**Authors:** Yu Wang, Maolin Zhou, Xiaochu Wu, Yanyan Wang

**Affiliations:** West China Hospital, Sichuan University, Chengdu, Sichuan, China (People’s Republic); West China Hospital, Sichuan University, Chengdu, Sichuan, China (People’s Republic); West China Hospital, Sichuan University, Chengdu, Sichuan, China (People’s Republic); West China Hospital, Sichuan University, Chengdu, Sichuan, China (People’s Republic)

## Abstract

Sarcopenia, a progressive age-related condition that is difficult to diagnose, increases the risk of adverse outcomes, including falls, frailty, and Alzheimer’s disease in older adults.Existing prediction models are limited by small sample sizes, difficult-to-collect predictors, and short prediction horizons. This study aimed to develop and validate a 6-year sarcopenia risk prediction model using routinely available clinical data.We employed a prospective cohort design, analyzing data from the West China Health and Aging Trend (WCHAT) study (baseline in 2018, 6-year follow-up in 2024), The baseline sample comprised 1,184 participants without sarcopenia, and data were collected on demographics, physical examinations, questionnaires, and laboratory tests. A support vector machine (SVM) model was developed, and sarcopenia was diagnosed according to the 2019 Asian Working Group for Sarcopenia (AWGS) criteria.During the 6-year follow-up, 78 participants (6.6%) developed sarcopenia.The final model included 19 predictors: age, weight, height, BMI, tooth count, toothbrushing frequency, waist-to-hip ratio, skeletal muscle mass, usual gait speed over 3 meters, mid-upper arm circumference, height-to-arm-circumference ratio, calf circumference, height-to-calf-circumference ratio, waist circumference, hip circumference, mobile phone use, fasting insulin, and HDL.Model validation showed a sensitivity of 0.71 (95% CI: 0.49–0.83), specificity of 0.78 (95% CI: 0.77–0.86), and AUC of 0.84 (95% CI: 0.77–0.90), respectively.This model, based on easily accessible clinical variables, showed good predictive performance for 6-year sarcopenia risk, indicating its potential clinical utility for identifying high-risk individuals who may benefit from closer monitoring or early intervention.

